# Application of Artificial Neural Networks to Predict the Intrinsic Solubility of Drug-Like Molecules

**DOI:** 10.3390/pharmaceutics13071101

**Published:** 2021-07-20

**Authors:** Elena M. Tosca, Roberta Bartolucci, Paolo Magni

**Affiliations:** Department of Electrical, Computer and Biomedical Engineering, University of Pavia, Via Ferrata 5, I-27100 Pavia, Italy; elenamaria.tosca@unipv.it (E.M.T.); roberta.bartolucci@unipv.it (R.B.)

**Keywords:** artificial neural networks, machine learning, QSPR, intrinsic aqueous solubility

## Abstract

Machine learning (ML) approaches are receiving increasing attention from pharmaceutical companies and regulatory agencies, given their ability to mine knowledge from available data. In drug discovery, for example, they are employed in quantitative structure–property relationship (QSPR) models to predict biological properties from the chemical structure of a drug molecule. In this paper, following the Second Solubility Challenge (SC-2), a QSPR model based on artificial neural networks (ANNs) was built to predict the intrinsic solubility (*logS*_0_) of the 100-compound low-variance tight set and the 32-compound high-variance loose set provided by SC-2 as test datasets. First, a training dataset of 270 drug-like molecules with *logS*_0_ value experimentally determined was gathered from the literature. Then, a standard three-layer feed-forward neural network was defined by using 10 ChemGPS physico-chemical descriptors as input features. The developed ANN showed adequate predictive performances on both of the SC-2 test datasets. Benefits and limitations of ML approaches have been highlighted and discussed, starting from this case-study. The main findings confirmed that ML approaches are an attractive and promising tool to predict *logS*_0_; however, many aspects, such as data quality, molecular descriptor computation and selection, and assessment of applicability domain, are crucial but often neglected, and should be carefully considered to improve predictions based on ML.

## 1. Introduction

Machine learning (ML) is a branch of artificial intelligence (AI) that automatically learns and finds hidden patterns from available knowledge and uses these patterns to make predictions on new data. Its application is strongly established in many research fields, and is also becoming popular in drug discovery and development, especially for the prediction of in vivo properties of new molecules. An excellent example is given by computational models based on quantitative structure–property relationships (QSPRs), in which descriptors accounting for molecule structure are used to predict physico-chemical properties, such as aqueous solubility. Aqueous solubility is one of the limiting factors to in vivo drug dissolution and, consequently, to absorption. For this reason, it is considered as a key physico-chemical parameter in drug discovery [1]. Poor solubility has been identified as a relevant cause of drug-development failures, and improving the aqueous solubility of bioactive molecules is a major issue in medicinal chemistry [2,3].

Aqueous solubility, *S*_w_, can be defined as the amount of drug (solute) that dissolves in a given volume of water (solvent) at a specified pH, temperature, and pressure. However, intrinsic aqueous solubility, *S*_0_, is typically considered in its place, in order to obtain a more reproducible measure that does not depend on pH. Indeed, *S*_0_ is defined as the solubility of a compound in its free acid or free base form. Several methods are available to experimentally determine the intrinsic aqueous solubility of a compound, such as variations of the saturation shake-flask method [4] and, more recently, the CheqSol (Chasing Equilibrium Solubility) techniques [5]. However, the experimental solubility determination proved to be difficult, time-consuming, and too expensive, or simply unrealistic to be applied in high-throughput screening, in which millions of compounds are tested. For this reason, in silico prediction of *S*_0_ has been widely used in the early stage of the drug discovery and development process.

One of the first and most popular methods to predict *S*_0_ is the general solubility equation (GSE) proposed by Yalkowsky [6,7]. Just two molecule characteristics, the octanol–water partition coefficient (*logP*) and the Celsius melting point (*T_mp_*), both experimentally determined, are used to estimate the *S*_0_ value in log molar unit (*logS*_0_):

(1) $$logS_{0}=0.5-logP-0.001\times \left(T_{mp}-25\right)$$

Due to its simplicity, GSE became the gold standard for the in silico prediction of *S*_0_. However, its reliance on experimental parameters such as melting point, the experimental measurement or calculation of which is as challenging as solubility, limits the GSE’s applicability, especially for virtual compounds (i.e., compounds developed in silico and not yet synthesized).

Predictive models based on QSPRs demonstrated to be promising tools to determine the solubility of drug-like molecules. In the last decades, a plethora of QSPR models based on ML, such as random forests (RFs), support vector machines (SVMs), partial least squares (PLS), k-nearest neighbors (k-NN), and artificial neural networks (ANNs), was proposed. Among these, ANNs were one of the most frequently proposed methods, demonstrating good predictive performances. Despite the numerous documented applications, the best models available in the literature were able to predict *logS*_0_ with a root-mean-square error (RMSE) of 0.7–1.1 log unit. Possible causes of the poor predictive performances of the available QSPR models are the subject of an intense debate in the scientific community, and are also discussed in this paper.

A primary source of this prediction error was identified in the poor reliability of experimental solubility values. Indeed, the quality of QSPR models is directly influenced by the quality of the datasets on which they are built. For drug-like molecules, the inter-laboratory variability, derived by comparing published intrinsic solubility values, is generally estimated to be 0.6–0.7 log unit or higher [8,9,10,11], even if Avdeef showed that with a critical curation of the sources, it could be reduced up to 0.17 log unit [12]. On the contrary, other studies suggest focusing attention on the improvements of the computational methods and on an accurate selection of the molecule descriptors used by such methods [8].

In order to engage the scientific community to address the issue of S_0_ prediction, two solubility challenges, named the First Solubility Challenge (SC-1) and the Second Solubility Challenge (SC-2), were organized by Llinas and Adveef in 2009 and 2019, respectively [13,14]. These challenges had two primary objectives: to assess the current state of the field and to provide recommendations on the best strategies to apply when making predictions. SC-1 asked participants to predict the intrinsic aqueous solubility of 32 drugs using a provided training dataset of 100 *S*_0_ values, all measured using the CheqSol method by the same group. On the contrary, in the SC-2 two test datasets, a 100-compound tight set composed by low variance and a 32-compound loose set of high-variance *S*_0_ values were provided. In SC-2, a common and standardized training dataset was not provided, and participants were invited to collect their own training set, providing references to the selected data sources.

The work here presented originates from SC-2 [14,15], with the main objective of investigating the use of ML approaches on an open research field, to highlight the benefits and limitations of these techniques on a specific case study and to show and discuss the fundamental steps necessary to develop an ML predictive model. Within this scope, we used an ANN, trained on a dataset we built from literature sources, to predict the *S*_0_ values of the 100-compound and 32-compound test sets of the SC-2.

## 2. Materials and Methods

### 2.1. Datasets

#### 2.1.1. Training Dataset

A dataset of known *S*_0_ for drug-like molecules was collected from the literature. The criteria adopted regarding whether to include a source was based on the recommendations reported in [14]. A list of possible reliable references was provided by Llinas et al. to support the participants new to the field [14]. Among these, the following 11 sources listing intrinsic solubility values were considered: Avdeef et al. (2000 and 2001) [16,17], Bergström et al. (2002, 2004a, and 2004b) [18,19,20], Sköld (2006) [21], Wassvik (2006) [22], 2008 Solubility Challenge [13,23], DLS-100 [24], and Baek (2018) [25]. All the included references reported experimental *S*_0_ measured using SSF and CheqSol techniques at about 25 °C. The list of compounds from the SC-1 [13,23] included four molecules for which two polymorphic forms were identified; for the purpose of this analysis, both the forms were considered as replicated values of the same molecules. In addition, five compounds were too soluble to be measured and two were decomposed during analysis; thus, they were not considered.

In the references, compounds were often listed by non-standardized names, and several synonyms for the same molecules were reported. To overcome this issue, CAS numbers were retrieved from the compound names either through a ChemIDPlus query (https://chem.nlm.nih.gov/chemidplus/ accessed on 22 February 2021) performed in R (*ci_query()* function of the R-package “webchem”), or via manual search. All the molecules for which a CAS number was not available were excluded.

In addition, solubility data were reported in many concentration units, either in natural or logarithmic scale. All the values were converted in molarity (mol/L) and tabulated in logarithmic unit, according to the SC-2 datasets [15]. To convert solubility values from the practical units (e.g., μg/mL) to molarity, the molecular weight had to be retrieved. First, for each compound, the Pubchem CID was obtained from the CAS number through the *get_cid()* R-function (R-package “webchem”), or via manual search. Then, the *pc_prop()* R-function (R-package “webchem”) was exploited to retrieve the molecular weights from the CIDs.

Since multiple values of intrinsic solubility collected from different sources were available for several molecules, alternative approaches to treat replicated experimental data were evaluated. Considered possibilities included taking the arithmetic average or the median of the experimental values, listing all the available replicates, or picking a single most-trusted value.

#### 2.1.2. Test Sets

The developed model was tested on the 100-compound low-variance tight set (test set 1) and the 32-compound high-variance loose set (test set 2) provided by the SC-2 [15], and detailed in Tables S2 and S3. These test sets were created by the SC-2 organizers with the specific purpose of challenging the ML methods and assessing their performances in contexts with different degrees of difficulty.

The two sets of test compounds were gathered from the 870 molecules included in the database Wiki-pS_0_ [12], the largest curated intrinsic solubility database known. Set 1 was composed of 100 drug-like molecules, the *logS*_0_ of which had an inter-laboratory standard deviation, SD_inter-lab_, ranging from 0.11 to 0.22 log unit [14], with an average value of 0.17 log unit (low-variance tight set). *LogS*_0_ fell in the interval (−6.79, −1.18) with a mean = −4.03 and an inter-compound SD, SD_test1_, of 1.27 log unit.

The 32 compounds of set 2 were the molecules with the highest SD_inter-lab_ of the Wiki-pS_0_ database, ranging from 0.50 to 0.93 log unit, with an average SD_inter-lab_ = 0.62 log unit (high-variance loose set). Compared to test set 1, test set 2 was characterized by a wider *logS*_0_ range, (interval_test2_ = (−10.4, −1.24), mean_test2_ = −5.49, and SD_test2_ = 2.18 log unit), with the majority of drugs having intrinsic solubility lower than 1 μM. This low solubility was recognized as the possible main reason for the poor overall reproducibility of experimental *logS*_0_ values [14]. Furthermore, several of these molecules (e.g., amiodarone, clofazimine, and itraconazole) were located in a sparsely populated chemical space, with very few nearby known similar molecules. For these reasons, an accurate prediction of their solubility was expected to be challenging.

For each test compound, the CAS number and Pubchem CID were obtained. Based on the CAS identifier, all the test molecules found in the training set were obviously removed, as specified in the SC-2 guidelines [14,15].

### 2.2. Structure Generation and Descriptor Calculation

Canonical SMILES (simplified molecular-input line-entry system) used to represent the molecular structure of the considered compounds (for both the training and test sets) were retrieved from the Pubchem CID via the *pc_prop()* R-function and submitted to a manual check.

SMILES strings were then used to calculate 35 topological and physico-chemical 2D descriptors. This was performed through the ChemGPS-NP (Chemical Global Positioning System—Natural Products) tool [26], available online at https://chemgps.bmc.uu.se (accessed on 14 May 2021). ChemGPS-NP uses the proprietary DragonX tool [27] as the internal engine for the calculation of hundreds of molecular descriptors, from which 35 were selected. Descriptor information is reported in [28].

Descriptors with zero inter-compound variance were removed. In addition, any compound of the training set with an undefined value for at least one of the descriptors was excluded from the final dataset. The presence of missing values was evaluated also for the test compounds.

Considering the limited number of data available in the training set, the high correlation between the obtained descriptors [29], and the fact that ANNs are sensitive to redundant information [30,31], a feature selection was necessarily performed to reduce the probability of reaching local minima and improve the ANN’s generalization performances. One of any pair of descriptors whose absolute correlation coefficient was greater than 0.8 was removed, retaining the descriptor with a higher absolute correlation with *logS*_0_ [12,32]_._ The resulting chemical space was further reduced by selecting the 10 most-relevant descriptors according to their correlation scores with the *logS*_0_.

Each descriptor d was scaled into the range (0, 1) according to:

(2) $$d_{\left[0,1\right],i}=\frac{d_{i}-min_{j=1\ldots N}\left\{d_{j}\right\}}{max_{j=1\ldots N}\left\{d_{j}\right\}-min_{j=1\ldots N}\left\{d_{j}\right\}}$$

where $d_{i}$ and $d_{\left[0,1\right],i}$ are the value of d for the ith compound, respectively, before and after the scaling; and $max_{j=1\ldots N}\left\{d_{j}\right\}$ and $min_{j=1\ldots N}\left\{d_{j}\right\}$ are the maximum and minimum values of d across all the N compounds of the training set, respectively. *LogS*_0_ values were scaled into the range (0, 1) in the same way [33]. To ensure that no data from the test sets were used into the model development and in the prediction steps, both descriptors and *logS*_0_ values of the two test datasets were scaled using the maximum and minimum values found in the training set.

### 2.3. Model Development: Artificial Neural Network (ANN)

The ANN model was developed using the multi-layer perceptron (MLP) algorithm with backpropagation contained within the Orange software [34]. *LogS*_0_ was considered as the target variable, and the 10 selected descriptors (see the previous section) as independent variables (features). The architecture of the network consisted of: (i) 10 neurons in the input layer, which corresponded to the 10 scaled descriptors selected from the correlation analysis; (ii) a hidden layer whose number of neurons was varied and selected based on the performances scores computed in the training dataset; and (iii) the output layer with one neuron, i.e., the scaled *logS*_0_. The regulation term α (L2 penalty) was tuned manually, and a logistic activation function was used for all the neurons in all the layers. Finally, the weight optimization was performed on the training dataset through the Adam solver for a maximum of 4000 iterations.

In order to select the best architecture, a cross-validation method was applied by splitting the training test in 10 groups (folds), using each of the folds in turn to validate the ANN, trained on the remaining 9 folds. The selected ANN was then trained again upon the entire training dataset and used to predict intrinsic solubility of molecules for the two SC-2 test datasets.

### 2.4. Model Performance Evaluation

To compare our results with the SC-2 findings, the same statistical measures of prediction performances (MPPs) were considered. These metrics included the *R*^2^, RMSE, bias (Equations (3)–(5)), and the percentage of predicted values within 0.5 log unit (denoted with % ± 0.5 log), which were computed using the following formula:

(3) $$R^{2}=1-\frac{\sum_{i=1}^{n}{\left(y_{i}-y_{i,pred}\right)}^{2}}{\sum_{i=1}^{n}{\left(y_{i}-\bar{y}\right)}^{2}}$$

(4) $$\mathrm{RMSE}=\sqrt{\frac{\sum_{i=1}^{n}{\left(y_{i}-y_{i,pred}\right)}^{2}}{n}}$$

(5) $$\mathrm{bias}=\frac{\sum_{i=1}^{n}\left(y_{i}-y_{i,pred}\right)}{n}$$

where *n* is the number of compounds in the considered dataset; $y_{i}$ and $y_{i,pred}$ are the experimental and predicted *logS*_0_ of the *i*th compound, respectively; and $\bar{y}$ is the average experimental value. These four metrics were computed and reported for the training dataset, the 10-fold cross validation of the training dataset, and the two test datasets. No compounds were removed as outliers; however, potential outliers, defined as data points with an absolute prediction error greater than a 2-fold RMSE, were further investigated to determine if their features deviated significantly from the rest of the dataset. In addition, the predictive performances of the ANN model for the two test datasets were compared with the ones reported by Llinias et al. for the GSE model [15].

Finally, the ANN performances were compared with the correspondent MPPs of the simplest and naïve prediction model (null model), in which every compound of the test datasets were predicted using the mean *logS*_0_ value computed on the training dataset ($\bar{logS_{0,train}}$). Given a training dataset, every model performing better than this “predict-average-for-all” model was considered a useful predictor.

### 2.5. Applicability Domain Assessment

An attempt to evaluate the relationship between the applicability domain coverage and the predictive performance of the model was performed. The domain of applicability (DOA) associated with our training set and dictated by the 10 selected descriptors (normalized within (0, 1)) was identified using two different approaches, based on PCA and a similarity measure.

A PCA was performed on training data, and the 100-compound test set 1 and the 32-compound test set 2 were projected on the obtained principal components (PCs). In particular, the two most important PCs (PC1 and PC2) were considered, and their 95% confidence interval was computed on the training data. This approach draws an ellipse in the 2-dimensional space defined by PC1 and PC2. Test compounds that fell outside this ellipse were considered poorly represented by the training data, and therefore were expected to be more difficult to predict.

In addition, an index of similarity (“normalized Euclidean similarity”) between compounds was defined by computing the Euclidean distance on the 10 normalized descriptors and dividing that distance by the maximum theoretical Euclidean distance (i.e., $\sqrt{10})$ to obtain a normalized score within (0, 1). Then, the similarity index was defined as the complementary of the normalized Euclidean distance, with 0 representing complete dissimilarity and 1 complete similarity. Thus, the similarity index between compound A and B, $Sim_{Index,A-B},$ was defined as:

(6) $$Sim_{Index,A-B}=1-\frac{\sqrt{\sum_{i=1}^{10}\left(d_{A,i}{}^{2}-d_{B,i}{}^{2}\right)}}{\sqrt{10}}$$

## 3. Results

### 3.1. Datasets and Descriptors

After the CAS query, the dataset contained 586 values of intrinsic solubility for 357 different molecules obtained from the 11 selected literature sources [13,16,17,18,19,20,21,22,23,24,25]. The comparison of our data with the published SC-2 test datasets revealed 57 compounds in common with the 100-compound test set 1 and 15 with 32-compound test set 2. Their removal from the training dataset left 430 *logS*_0_ values for 285 different molecules.

For each of the 285 compounds, SMILES strings were submitted to ChemGPS and the list of 35 descriptors [28] was retrieved. One descriptor, presenting the same values for all the 285 molecules, was excluded from the analysis. In addition, 15 compounds with an undefined value of at least one of the remaining 34 descriptors were removed. This resulted in a total of 412 *logS*_0_ values for 270 different molecules, with 34 usable descriptors for each.

**Figure 1:**
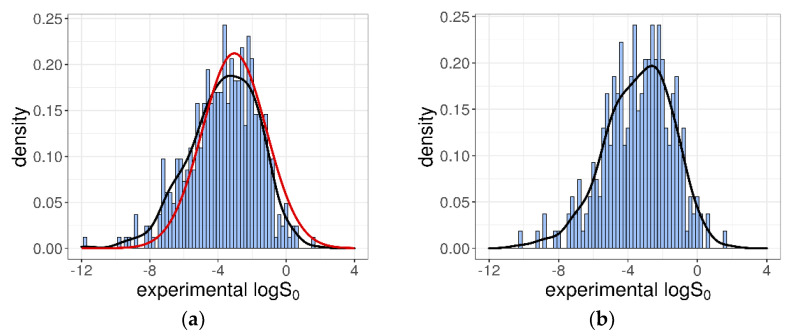
(**a**) Distribution for the collected 412 intrinsic solubility entries. The red line represents the Gaussian distribution for the 6355 solubility entries of Wiki-pS_0_ database. (**b**) Intrinsic solubility distribution for the 270-compound training dataset composed of the average *logS*_0_ values.

The 412 intrinsic solubility values ranged from −11.76 to +1.7 log molarity, with about 53% of them falling between −7 and −3 log unit, which corresponded to the typical range for drugs and research compounds [35]. The *logS*_0_ distribution was essentially a Gaussian characterized by a mean = −3.66, a median = −3.52, and a SD = 2.02 log unit. In Figure 1a, the obtained distribution is shown and compared with the distribution of the *logS*_0_ values collected in the Wiki-pS_0_ database [12]. From this, it is evident that, even if the number of solubility entries was significantly lower (412 vs. 6355), our dataset was representative of the more comprehensive Wiki-pS_0_ database, at least in terms of *logS*_0_ values. However, because the whole collection of Wiki-pS_0_ molecules is not publicly available, the similarity in the “chemical feature space” with our training dataset cannot be evaluated.

As summarized in Table 1, there were 81 different molecules for which solubility was reported from at least two different sources. Based on these 81 replicated values, the average inter-laboratory standard deviation, SD_inter-lab_, was determined to be 0.78 log unit. This value was comparable to the experimental reproducibility suggested in previous studies (0.6–0.7 log unit [8,9,10,11]), but was significantly greater than the 0.17 log unit value estimated by Avdeef [12]. The observed difference in the SD_inter-lab_ likely was due to the wide range of the *logS*_0_ values reported in literature for some molecules.

According to the SC-2 test datasets, the training dataset was built considering the inter-laboratory average values by computing the arithmetic mean of the duplicated *logS*_0_ values. The training dataset was thus composed of 270 different molecules, each reported with its average *logS*_0_. The *logS*_0_ values fell within the interval (−10.26, +1.7) log unit, and were characterized by a mean = −3.4 and an SD = 1.95 log unit, as shown in Figure 1b.

For both the test datasets, the ChemGPS descriptors were computed by using the previously retrieved SMILES strings. No missing values were found in the descriptors of the two test sets.

### 3.2. Features Selection

With the aim of reducing redundant information and improving the ANN performances, the highly correlated descriptors (absolute correlation coefficient >0.8) were removed. The remaining 18 descriptors were ranked according to their correlation with the *logS*_0_ values of the training dataset, and only the first 10 were selected as input features for the ANN. Table 2 lists the selected descriptors and their correlation coefficients with *logS*_0_. As expected, the best (negative) correlation was achieved with the ALOGP descriptor, with a value of −0.637. Descriptor values for the 270-compound training dataset and for the two test datasets are reported in Tables S1–S3, respectively. More information about their meaning can be found in [27,28].

### 3.3. Artificial Neural Network

Several ANN models with different numbers of neurons in the hidden layer and regulation term α were generated with the Orange platform. The optimization of the architecture was performed with the 10-fold cross-validation of the training dataset, selecting the best structure in terms of *R*^2^ and RMSE. The final ANN structure, with 25 neurons in the hidden layer and α = 0.2, showed *R*^2^_cross_ = 0.51, RMSE_cross_ = 1.37, bias_cross_ = −0.027, and %0.5 ± log_cross_ = 33.

**Figure 2:**
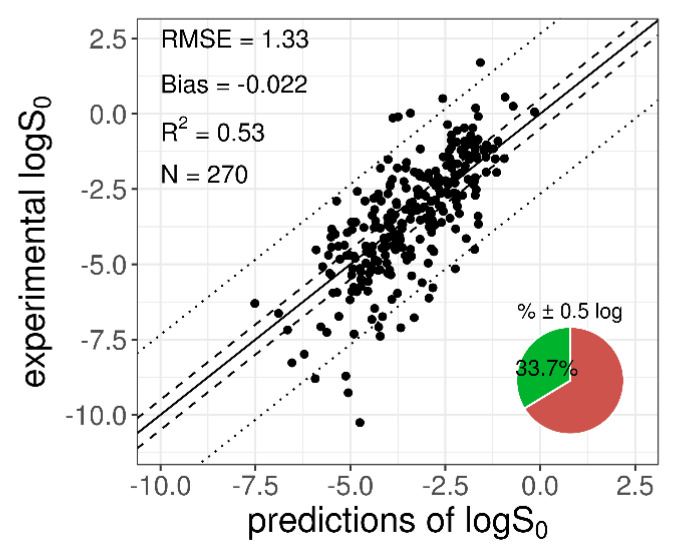
Plot of the experimental versus predicted *logS*_0_ for the 270-compound training dataset. The solid diagonal line represents the identity line; dashed and dotted lines represent the displacement from the identity line by ±0.5 log and by ±2 × RMSE log, respectively. The pie chart refers to the percentage of “correct” predictions (within % ± 0.5 log).

When the entire training dataset (*n* = 270) was used to train the selected network (with a fixed structure), the resulted MPPs were: *R*^2^_train_ = 0.53, RMSE_train_ = 1.33, bias_train_ = −0.022, and %0.5 ± log_train_ = 33, confirming the results obtained in the cross-validation step (Table 3). A plot with the *logS*_0_ experimental and predicted values for the training dataset is shown in Figure 2.

**Figure 3:**
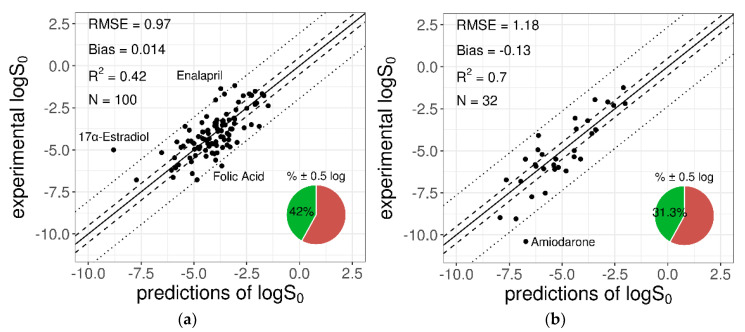
Plots of the experimental versus predicted *logS*_0_ for the 100-compound low-variance tight set (**a**) and the 32-compound high-variance loose set (**b**) provided by the SC-2. The solid diagonal line represents the identity line; dashed and dotted lines represent the displacement from the identity line by ±0.5 log and ±2×RMSE log, respectively. The pie chart refers to the percentage of “correct” predictions (within % ± 0.5 log). Names of outlier compounds are displayed.

**Figure 4:**
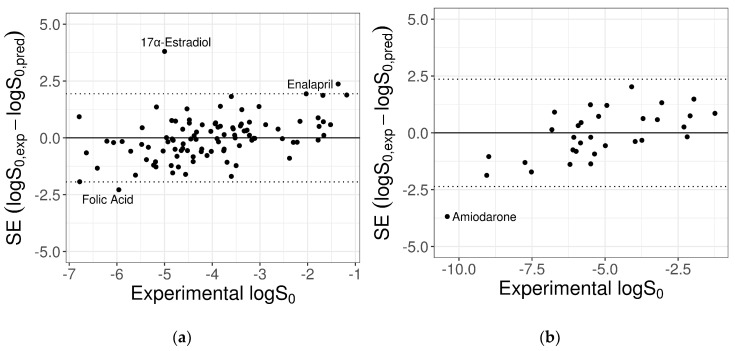
Plots of the SE versus experimental *logS*_0_ for the 100-compound low-variance tight set (**a**) and the 32-compound high-variance loose set (**b**) provided by the SC-2. Dotted lines mark the cut-off threshold for outliers equal to 2×RMSE (test 1: 2.32 log unit; test 2: 2.48 log unit). Names of outlier compounds are displayed.

The trained ANN was used to predict the *logS*_0_ values for the 100-compound test set 1 and the 32-compound test set 2 provided by the SC-2. The MPPs computed on the two test datasets are reported in Table 4, together with the same metrics obtained with the GSE formula and the null model. Figure 3 and Figure 4 show the experimental versus predicted solubility and the standard errors (SE) plotted against *logS*_0_, respectively. In addition, in Tables S2 and S3, the predicted values are given.

As shown in Table 4, the GSE and ANN performances were almost comparable for both the SC-2 test sets. Instead, to quantify the gain in performance introduced by the ANN model, the difference in terms of RMSE was computed between the ANN model and the null model, in which the average *logS*_0_ of the training compounds ($\bar{logS_{0,train}}$ = −3.4 log molar) was used as prediction for all the test compounds. A decrease of 0.44 log unit for test set 1 and of 1.659 log unit for test set 2 was observed, confirming that overall, the ANN model performed strongly better than a minimally useful predictor model, especially for the “highly variable” test set 2.

It is clear that the 100-compound tight set (test set 1) was generally better modelled in terms of absolute error measures such as RMSE, bias, and percentage of correct predictions within ±0.5 log unit. This was expected, since test 2 was created with the explicit purpose of being more challenging. However, accounting for the higher inter-laboratory uncertainty affecting the solubility values of the loose set (SD_inter-lab,test1_ = 0.17 log unit versus SD_inter-lab,test2_ = 0.62 log unit), the differences in the prediction performances for the two datasets were not so relevant: RMSE-SD_inter-lab,test2_ = 0.56 < RMSE-SD_inter-lab,test1_ = 0.80.

Similar observations resulted in accounting for the wider solubility range of the loose set (9.16 log unit span for test set 2 versus only 5.61 log unit for test set 1). Indeed, the increase in RMSE between test sets 1 and 2 was proportionally smaller than the increase in the inter-compound standard deviations in the two datasets (i.e., SD_test1_ = 1.27 log unit versus SD_test2_ = 2.18 log unit), such that the RMSE/SD ratio was significantly smaller for the loose set (RMSE/SD_test2_ = 0.54 < RMSE/SD_test1_ = 0.76). Accordingly, the loose set had a better *R*^2^.

Overall, there was a significant variation in the prediction accuracy between different test molecules, with better predictions for compounds with intermediate solubility values. In particular, the ANN model adequately predicted most of compounds, with very few exceptions for which the prediction error was particularly high (Figure 4). The impact of these compounds on the MPPs could be relevant, considering both their reported *logS*_0_ values and the reduced size of the test datasets.

For the 100-compound set, three molecules were identified as outliers; i.e., with an absolute prediction error greater than twofold RMSE (2 × RMSE = 1.94 log unit). From the worst, they were: 17α-estradiol, enalapril, and folic acid. The contribution of each of these to the MPPs was quantified by removing one compound at a time and re-computing the statistical metrics on the remaining molecules, as summarized in Table 5. The removal of the three outliers increased the predictive model performances for *R*^2^ from 0.42 to 0.54 and for RMSE from 0.97 to 0.84.

Similarly, outlier analysis of the 32-compound set showed that for only one molecule, amiodarone, the absolute SE exceeded the threshold 2×RMSE = 2.36 log unit. The exclusion of this compound provided a quite relevant gain of model MPPs, as reported in Table 6.

The possible causes of the poor model performances on these “outlier” compounds were investigated, with particular attention to the issue of applicability domain coverage.

### 3.4. Outliers and Applicability Domain

To assess the reliability of the solubility predictions and understand the possible causes of the poorly predicted values, the domain of applicability of the trained model as dictated by the training dataset was considered.

**Figure 5:**
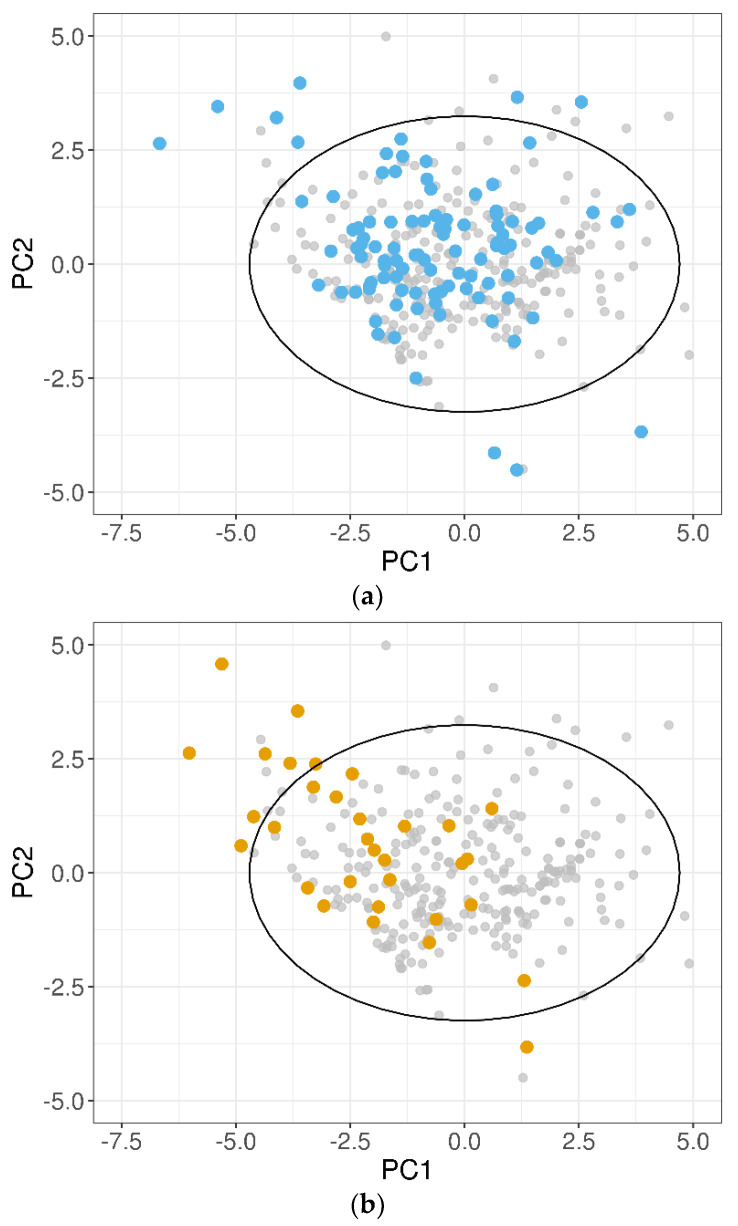
Projections of the (**a**) 100-compound test set 1 (blue dots) and (**b**) 32-compound test set 2 (orange dots) on the two most important PCs of the training dataset. Grey dots show the position of the training compounds. The black solid ellipse marks the 95% confidence interval of the training domain.

A PCA on the 10 normalized descriptors (Table 2) was performed for the training dataset, and the test sets were projected on the obtained PCs. In Figure 5, the 100-compound test set 1 (panel (a)) and the 32-compound test set 2 (panel (b)) were plotted in the two-dimensional space defined by PC1 and PC2 of the training dataset, which accounted for 53% of the total variance. It was evident that for both the test datasets, some compounds fell outside the 95% CI ellipse that identified the DOA. In addition, several test set 2 molecules, although inside the 95% CI ellipse, were placed in poorly representative areas of the training domain.

**Figure 6:**
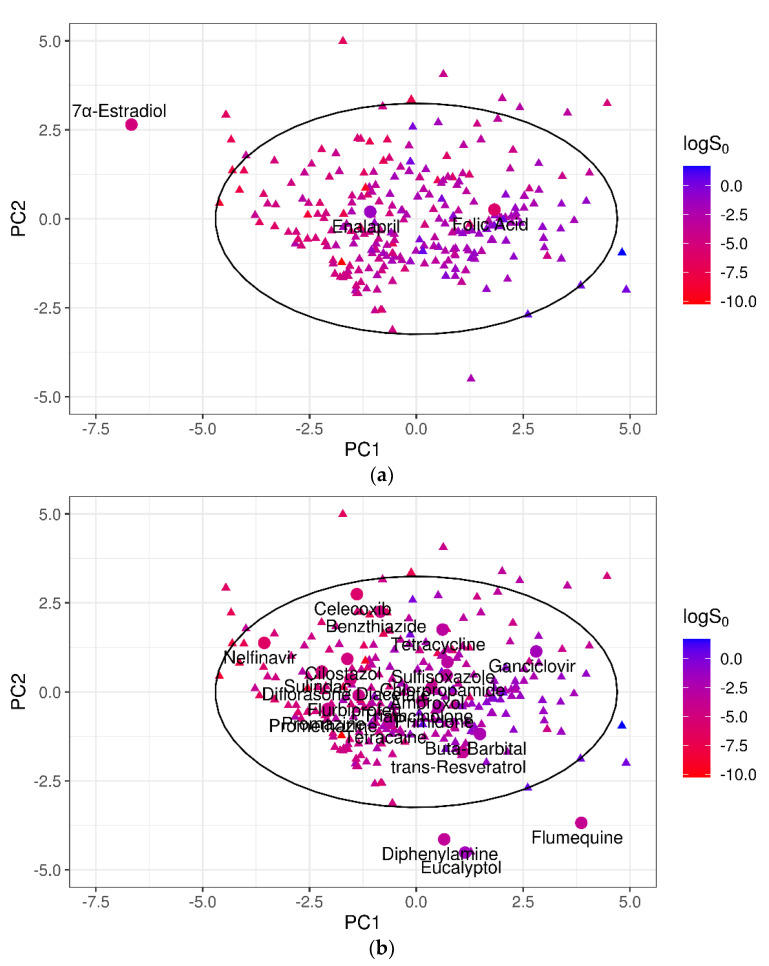
(**a**) Projection of the three worst-predicted outliers of test set 1 in the PC1–PC2 dimensional space. (**b**) Projection of the 22 best-predicted compounds of test set 1 in the PC1–PC2 dimensional space. Circles mark the test compounds; triangles represent the 270 training molecules; and the black solid ellipse marks the 95% CI of the training domain. For both training and test compounds, solubility value is represented by color.

To investigate the relationship between the model performances and the DOA coverage, the poorly predicted outliers were examined. For test set 1, one of the three worst-predicted compounds; i.e., 17α-estradiol, fell outside the 95% CI of the training domain (Figure 6a), suggesting that the prediction quality may deteriorate outside the DOA. To reinforce this hypothesis, the best-predicted compounds of test set 1 (i.e., with absolute SE ≤ 0.2 × RMSE = 0.20 log unit) were also considered. Nineteen of the 22 molecules thus identified clearly laid inside the training domain (95% CI ellipse), in particular in areas of the PC1–PC2 plane richly populated by training compounds (Figure 6b).

Using PCA as a basis for the DOA definition, 17α-estradiol was not represented by the training compounds. Hence, it was of little surprise to find that this compound was poorly predicted. On the contrary, two of the outliers, enalapril and folic acid, did fall within the 95% CI of PC1–PC2, and therefore their poor predictability could not be due to the DOA coverage issue. For both these molecules, a significant difference in the *logS*_0_ values was observed compared to those of their 10 nearest neighbors, which were identified by the similarity index that we introduced based on the normalized Euclidean distance of the 10 selected descriptors (Equation (6)). In particular, *logS*_0_ values of the folic acid neighborhood varied from −7.11 to −2.04, with a mean of −3.95 log unit. Accordingly, the predicted value for folic acid was *logS*_0_ = −3.68 log molar, almost identical to the average value of the 10 NN, but extremely different from the experimental *logS*_0_ (−5.96 log molar). Similarly, the experimental *logS*_0_ of enalapril was −1.36 log molar, even outside the range of its 10 NN, the solubility of which ranged from −5.9 to −2.9 log unit.

**Figure 7:**
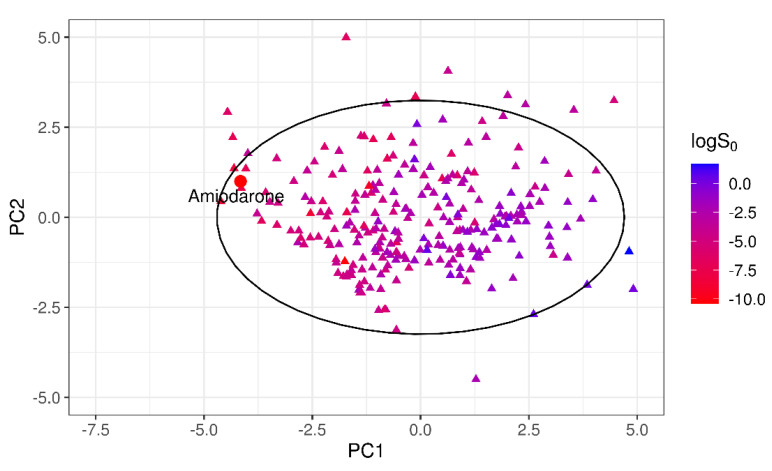
Projection of the test 2 outliers in the PC1–PC2 dimensional space.

The same DOA assessment was performed for the 32-compound test set 2. Figure 7 shows the position of the worst-predicted compound, amiodarone, in the PC1–PC2 plane.

Although inside the 95% CI of the training dataset, amiodarone clearly laid in a sparsely populated area of the training chemical space, as confirmed by the values of the Euclidean similarity index. As for the test set 1 outliers, the 10 nearest neighbors of amiodarone (*logS*_0_ = −10.4 log molar) based on the Euclidean similarity index were considered. They showed a high variance in terms of *logS*_0_, with a range of −9.27 to −0.1, and a mean of −4.53 log unit. The predicted amiodarone solubility was −6.72 log molar, barely higher than the mean solubility value of the 10 NN.

## 4. Discussion

Predicting the intrinsic solubility of drug-like molecules is of extreme relevance for a vast array of applications, among which is the prediction of in vivo dissolution. For this reason, any insight into possible strategies to improve solubility prediction are of significant interest. The release of the SC-2 results [15] created an opportunity to investigate the possible contribution of ML techniques in this area. Embracing the SC-2 as non-competitive participants, in this study an ANN model was developed based on literature-harvested *logS*_0_ data, and it was used to predict the intrinsic solubility value for two given SC-2 test datasets of drug-like molecules.

Intrinsic solubility values for 270 drug-like molecules were collected from a list of literature sources, and a training dataset was composed based on the inter-laboratory variability principle, thus computing the average *logS*_0_ of the available replicates. On this training dataset, a standard three-layer feed-forward neural network was developed using 10 ChemGPS-NP physico-chemical descriptors as input features. The developed ANN demonstrated adequate predictive performances on both the 100-compound low-variance tight set (test set 1) and the 32-compound high-variance loose set (test set 2) provided by SC-2 as test datasets, with an RMSE of 0.97 and 1.18 log unit for test set 1 and 2, respectively. Model predictive ability further improved for a reduced subset of test molecules that excluded very few poorly predicted outliers, reaching RMSE = 0.84 and RMSE = 1.00 log unit for a 97-compound subset of test 1 and for a 31-compound subset of test 2, respectively.

Our results were comparable with the most competent models submitted to the SC-2. The average RMSE over the 37 models submitted to the SC-2 was approximately 1.1 and 1.58 log unit for the low-variance and high-variance test datasets, respectively. Considering only predictors based on neural networks (30% of the submissions), the RMSE mean was approximately 1.45 and 1.87 log unit for test set 1 and 2, respectively. The ANN model that performed overall better on both the datasets obtained an RMSE of 0.93 and 1.24 log unit for test set 1 and 2, respectively, equivalent to our results.

According to the SC-2 findings [15], in absolute terms (i.e., RMSE, bias, and % ± 0.5 log) the low-variance tight set was better predicted than the high-variance loose set. Solubility of inconsistently determined molecules (high SD_inter-lab_), especially of poorly soluble molecules from sparse areas of chemical space, was more difficult to predict compared to the consistently determined solubility (low SD_inter-lab_) of compounds from well-represented parts of the drug-like domain. This observation suggests that “some test sets are harder to model than others”, as concluded by Mitchell et al. [32]. However, when the average errors of each dataset (SD_inter-lab_) were considered, model predictive accuracies on test set 1 (low SD_inter-lab_) and test set 2 (high SD_inter-lab_) were about the same. For this reason, it is not possible to unambiguously discriminate the contribution of the experimental data quality (low versus high SD_inter-lab_) and of the training domain coverage to the different predictive capability of the model on test set 1 and test set 2 compounds.

Overall, the results of this work were aligned with the conclusions of the SC-2. The adoption of sophisticated ML techniques, such as ANNs, did not provide superior benefits to the simpler modeling approaches such as GSE, which performed as well as more complex models. However, the usefulness of the ANN architecture was clearly demonstrated. Indeed, given a training dataset, a minimally useful predictor is defined as a model performing better than the “predict-average-for-all” model (null model). The ANN model proposed in this study outperformed the null model, showing that the prediction quality of the neural network was substantially better than a minimally useful predictor.

The use of QSPR models based on ML techniques appears to be an attractive approach that could provide relevant contributions to the solubility field. However, based on our experience, the following recommendations are of paramount importance during the development of a ML model to predict intrinsic solubility for drug-like molecules, and more in general when ML techniques are used.

### 4.1. Consideration of the Data Quality

Prediction accuracy of computational QSPR models is strongly and directly influenced by the quality of the data. Thus, knowledge regarding the reliability of data is fundamental in acknowledging the limitations of any subsequent computational data-driven predictor.

Experimental measurement of *S*_0_ is not an easy task to perform, and several factors, such as temperature, physical form of the precipitate, solution pH, and ionization state, as well as the presence of different tautomeric forms (which may have different physico-chemical properties) in equilibrium in the solutes, can contribute to its variability [36,37]. As a result, while taking care to select reliable sources for solubility data, unidentified errors due to mistakes and variability in the experimental methodologies would be undoubtedly introduced. For a significant number of drug-like molecules, inconsistent intrinsic solubility values are reported in the literature. Despite Adveef determining the average inter-laboratory reproducibility of 870 molecules from the Wiki-pS_0_ database as 0.17 log unit [12], different studies in the literature and our findings suggested that the typical error of reported intrinsic solubilities of drug-like molecules is around 0.6–0.7 log unit [8,9,10,11]. Because the accuracy of a model cannot exceed the accuracy of the experimental data, this implies that the best realistically possible predictor would achieve an RMSE similar to the inter-laboratory standard deviation of *logS*_0_ data; i.e., around 0.6–0.7 log unit.

If it is true that the quality of QSPR models is directly influenced by the quality of the datasets on which they are trained, it is equally true that the assessment of predictive performances of a model is strongly influenced by the accuracy of the data on which it is tested. Indeed, the observed performances derive from the contribution of the actual predictive performances (defined as the accuracy of a model that would be observed on a test set with zero internal error) and the uncertainty in the test data [38]. In the case of the intrinsic solubility, due to poor reliability of experimental solubility values, the observed performances could be significantly influenced by the errors affecting the *logS*_0_ values of the test compounds. For this reason, it is essential to carefully considered the internal error of the test data. The assessment of the proposed ANN against the 100-compound low-variance tight set and the 32-compound high-variance loose set, provided by SC-2 and characterized by a different internal error, was done exactly for this purpose.

### 4.2. Careful Curation of the Training Dataset

It is a well-known fact that increasing the number of data instances in the training set has a positive effect on the accuracy of data-driven models. At the same time, data should be congruent with respect to the problem statement. For example, for the intrinsic solubility case study, the inclusion of solubility data of organic compounds other than drug-like molecules, such as industrial organic molecules and agrichemicals (herbicides, pesticides, insecticides, rodenticides, and acaricides), could significantly increase the size of the training dataset. However, it was observed that the predictive performances of ML models developed on a training dataset including non-drug-like compounds were usually inadequate [15,39].

In addition, the use of training data harvested from the literature leads to the usual critical problem of combining data from many sources, which could be obtained under vary experimental conditions not always well documented in the original sources. The presence of replicated and often contradictory solubility values for the same compound highlights the issue of selecting an adequate strategy to manage them. Different approaches were discussed in the literature, such as computing the mean or the median of the replicates, or selecting either a single most-trusted data per molecule or else the average of only the replicates considered trustworthy [32]. Because the SC-2 test datasets were compiled based on inter-laboratory average values, our training dataset was built following the same strategy (arithmetic mean of *logS*_0_). However, the impact of alternative strategies to handle replicated solubility values was evaluated. Two different training datasets were built by including all the replicated *logS*_0_ values for each molecule (Training_replicates_) or taking their median (Training_median_). In both the cases, the number of neurons in the hidden layer and the value of the α regulation term was optimized with a 10-fold cross validation step. Twenty-five neurons and α = 0.2 provided the best performances on the training datasets. Interestingly, the performances of the obtained ANN models were comparable with only a slight deterioration on test 2 for the Training_replicates_ (Table 7). These results suggest that using the mean or median did not have a relevant impact on the model performances, and that including replicates of the same compounds did not improve or, in case of contradictory values, even reduced the performances of the ANN model.

### 4.3. Assessment of the Applicability Domain

The definition of a predictive domain of a model is a critical step to set up a data-driven model. ANNs, as well as other ML approaches based on the information encoded in the data, hardly extrapolate beyond the domain of their training dataset. Although predictions made outside of the DOA are not necessarily “wrong”, such predictions are considerably less reliable and should be treated with extreme caution. Therefore, the knowledge of the DOA is fundamental when making a prediction. In this work, the relationship between the DOA coverage and the predictive performance of the ANN was evaluated. The training domain was defined using tailored similarities based on PCA and Euclidean distance, and considering only the relevant descriptor selected for the model. From the PCA, it resulted that for both the test datasets, some compounds fell outside the training domain or were placed in very poorly populated areas. In addition, the position with respect to the DOA of the worst- (SE > 2 × RMSE) and best-predicted (SE < 0.2×RMSE) compounds was investigated. Of interest, the latter clearly laid in richly populated areas within the training domain. On the contrary, the poorly predicted outliers were not well represented by the training compounds. Indeed, some of them were completely outside the predictive domain of the model (i.e., 17α-estradiol) or in sparsely populated area (i.e., amiodarone); others, although inside the DOA, showed *logS*_0_ value extremely different from their neighborhood in the training dataset (i.e., folic acid and enalapril). In such situations, it was not surprising that poor predictions were obtained.

### 4.4. Selection of Input Features

Molecular descriptors provide a mathematical representation of the chemical information of a compound. A vast array of descriptors, coding for a plethora of properties (hydrophobicity, steric, hydrogen bonding, molecular flexibility, and electrostatic and topological interactions) can be computed from the SMILES representation via a multitude of software tools, both proprietary and freely available. All these descriptors are possible candidate inputs for QSPR models aimed to predict chemical properties of molecules, such as aqueous solubility. However, on one side, the large number of chemical descriptors could cause identification issues in the model-development process, while on the other side, the presence of redundant information due the notoriously high correlation of descriptors could lead to overfitting problems. Thus, it is usually preferred to use a modest number of relevant input features. Which descriptors could be the most relevant for the prediction of the intrinsic solubility of drug-like molecules is a point of considerable debate. In addition, the absence of descriptors coding for some physico-chemical property relevant to the solubility has been enumerated as one of the possible reasons for the poor predictive performance of the QSPRs. In this work, descriptors computed by ChemGPS-NP, a validated tool based on DragonX, were considered. ChemGPS-NP provides 35 molecular features that are selected from a total of 926 descriptors computed via DragonX in order to (1) provide descriptors with a comprehensible physical meaning (improving the explanation of chemical space), (2) distinguish between compounds, (3) encode relevant aspects of molecular complexity, and (4) describe important molecular properties such as lipophilicity, polarity, size/shape, hydrogen bond capacity, polarizability, flexibility, and rigidity [28].

Several software tools computing alternative sets of molecular descriptors are available and could be considered. In the preliminary phases of this work, the 1444 1D and 2D descriptors computed with the PaDEL-descriptor software [40] based on the Chemistry Development Kit (CDK) [41] have been evaluated without any improvements in the model performances (data not shown). Another frequently considered software tool is RDKit [42], the descriptors of which have been considered as input features by Avdeef [12] and some of the SC-2 participants. The results did not provide any evidence that the use of the consolidated RDKit descriptors improves the intrinsic-solubility predictions.

Due to the high correlation of the 35 ChemGPS-NP descriptors and the limited size of the training dataset, a further selection based on their correlation with solubility was performed, thus reducing the considered descriptors to a subset of 10 input features. Among them, the best (negative) correlation was achieved by ALOGP. This was expected, considering the historical relevance of the octanol–water partition coefficient, *logP*, in the prediction of intrinsic solubility. Indeed, *logP*, together with melting point, *T_mp_*, was one of the two variables composing the GSE. Due to the relevance of the GSE and, in turn, of experimental *logP* and *T_mp_* in the intrinsic-solubility prediction, few additional considerations of these two important molecular characteristics were undertaken.

First of all, in accordance with general accepted choices in solubility QSPR model development, a predicted value of *logP* was used in our analysis instead of the experimental value. In particular, we used the Ghose–Crippen ALOGP computed by ChemGPS-NP, which is one of the computational methods most widely applied to predict *logP* [43]. The ChemGPS-NP ALOGP and the experimental *logP* values (provided by the SC-2 organizers) of the 132 test molecules were compared to verify their consistency. The predicted and experimental *logP* were in good agreement (*R*^2^ = 0.87) with very few exceptions. Of interest, 17α-estradiol, one of the outliers of our ANN, had the worst predicted *logP*. After replacing the ALOGP value of 17α-estradiol with its experimental measure, an improvement of its solubility prediction (absolute prediction error decreased from 3.8 to 2.48 log unit) was observed. Moreover, considering that the octanol–water partition coefficient summarizes several molecular characteristics and is strongly related to the (polar) molecular surface area and the presence of polar groups, we explored the possibility of training a network in which the ALOGP input was replaced by some descriptors related to surface area and polarity [28]: topological polar surface area (TPSA (NO)); hydrophilic factor (Hy); and the counts for oxygen (nO), aliphatic/aromatic hydroxyl groups (nROH/nArOH), and nitrogen (nN), for a total of 13 input features. As shown in Table 8, predictive performances of the obtained model were worse than those obtained using the ALOGP descriptor.

A similar analysis was performed for the *T_mp_*. Because ChemGPS-NP does not provide *T_mp_*, it was not included into the list of the possible input features of the trained ANN. To evaluate if the availability of *T_mp_* could improve the ANN performance, the experimental melting points for 188 training compounds were subsequently retrieved from the CAS number by using the MPBPWIN module of EPI (estimation programs interface) [44]. The ANN was re-trained on these 188 molecules, both adding *T_mp_* or not to the 10 previously selected descriptors. Interestingly, when the obtained models were used to predict the compound solubility, they demonstrated comparable performances (Table 9).

Other aspects that are important to keep in mind during the selection of input features are the intrinsic limitations affecting the molecular descriptors computed from the SMILES representation. First, a single molecular structure can be represented by multiple SMILES [45]. It is thus essential to retrieve the canonical SMILES, which provides a unique string for each molecule. Second, the SMILES string (even in the canonical one) is a bi-dimensional approximation of the molecular structure that does not retain 2D or 3D coordinates for individual atoms. Therefore, the impact of the 3D structure on the physico-chemical properties, such as solubility, is not accounted for. Finally, the use of molecular descriptors computed from the SMILES is complicated by tautomerization. Indeed, some molecules can exist in several tautomeric forms, that may show different physico-chemical properties [46], and their equilibrium in the solutes is influenced by the experimental conditions. This information is rarely reported in the literature, making it impossible to know the tautomer to which the retrieved solubility value refers. Therefore, the choice of which tautomer has to be used to compute the descriptors could have a significant impact on the model’s prediction performances. Note that the canonical SMILES retrieved from PubChem used in this work refers only to one tautomeric form, which may not correspond to the one for which the available experimental *logS*_0_ was measured. Consequently, the accuracy of the QSPR model could be affected.

### 4.5. Interpretability and Reproducibility of the Results

The fact that ANNs and other ML approaches produce “black-box” models that are hard to interpret by humans is widely acknowledged. In addition, in a large number of published works, details on datasets, selected features, steps of the model building and parameters settings, and criteria for the evaluation of the results are often not fully provided and clearly explained. This lack of transparency significantly hampers the interpretability and the comparison of the results, even becoming the major problem in trusting these kinds of approaches. Reproducibility of the results should be one of the main aspects on which to focus future efforts.

For example, in both the Solubility Challenges, the participants were not asked to provide details about the computational methods they used, the molecular descriptors actually included as input features of their ML models, or any additional experimental data they employed. For these reasons, although SC-1 and SC-2 provided useful benchmarks to the solubility field, the results were difficult to interpret. Many actions could be taken to improve the understanding and reproducibility of the current computational methods; for example, the creation of open data sharing with the values of solubility for both the training and test compounds, as well as the molecular descriptors; a more transparent report of the adopted methodologies for features selection and ML algorithm; and finally, the adoption of standardized metrics for the evaluation of performances in the prediction of solubility for drug-like compounds.

## 5. Conclusions

The use of ML approaches to develop a predictive QSPR model was investigated in this work on the particularly challenging issue of the prediction of intrinsic solubility of drug-like molecules, with a primary objective of providing a detailed overview of the required steps and the main problems encountered when such task is performed.

The conclusion of the present work is that developing ML-based QSPR models to accurately predict intrinsic aqueous solubility of drug-like molecules is still a formidable challenge. Confirming the results of the Solubility Challenges, we highlighted that more high-quality solubility data and more discriminant descriptors are needed. Moreover, as some of the considered drugs illustrated, there are still under-populated neighborhoods in the chemical space of drug-like molecules.

In summary, the adoption of ML approaches to accurately predict intrinsic solubility is promising and attractive, but it needs to be further enhanced. Nonetheless, it is an issue that is still of great importance, with aqueous solubility being at the heart of pharmaceutical design.

## Figures and Tables

**Table 1:** Summary of the replicated values of training dataset.

Number of Molecules	Number of Replicated Values per Molecule
189	1
50	2
18	3
7	4
1	5
3	6
1	8
1	10

**Table 2:** List of the 10 selected ChemGPS descriptors.

ChemGPS Descriptors		Correlation Coefficient with *LogS*_0_
Abbreviation	Description	
ALOGP	Ghose–Crippen octanol–water partition coefficient	−0.637
nC	number of carbon atoms	−0.583
nCIC	number of rings	−0.511
nBnz	number of benzene-like rings	−0.510
Ui	unsaturation index	−0.434
Me	mean atomic Sanderson electronegativity (scaled on C atom)	0.310
RBN	number of rotatable bonds	−0.224
nN	number of nitrogen atoms	0.214
nX	number of halogens	−0.200
Hy	hydrophilic factor	−0.138

**Table 3:** Statistical MPPs obtained with the 10-fold cross validation and the entire training dataset.

Dataset	*R* ^2^	RMSE	Bias	% ± 0.5 Log
10-fold cross validation	0.51	1.37	−0.027	33
Entire training dataset	0.53	1.33	−0.022	34

**Table 4:** Statistical measures of prediction performances obtained for the SC-2 test datasets.

Model	100-Compound Test Set 1				32-Compound Test Set 2			
	*R* ^2^	RMSE	Bias	% ± 0.5 Log	*R* ^2^	RMSE	Bias	% ± 0.5 Log
ANN	0.42	0.97	−0.014	42	0.70	1.18	−0.133	31
GSE	0.22	1.12	−0.29	41	0.69	1.20	−0.073	22
Null model	−0.25	1.41	−0.63	29	−0.74	2.83	−1.84	13

**Table 5:** Statistical MPPs obtained on test set 1 after removal of outliers.

Removed Compounds	*R* ^2^	RMSE	Bias	% ± 0.5 Log
None (full test set 1)	0.42	0.97	−0.014	42
17α-Estradiol	0.51	0.89	−0.024	42
17α-Estradiol, Enalapril	0.52	0.87	−0.049	43
17α-Estradiol, Enalapril, Folic Acid	0.54	0.84	−0.025	43

**Table 6:** Statistical MPPs obtained on test set 2 after removal of outliers.

Removed Compounds	*R* ^2^	RMSE	Bias	% ± 0.5 Log
None (full test set 2)	0.70	1.18	−0.133	31
Amiodarone	0.74	1.00	−0.019	32

**Table 7:** Statistical measures of prediction performances obtained on the SC-2 test sets with ANN based on Training_median_ and Training_replicates_ datasets.

Training Datasets	100-Compound Test Set 1				32-Compound Test Set 2			
	*R* ^2^	RMSE	Bias	% ± 0.5 Log	*R* ^2^	RMSE	Bias	% ± 0.5 Log
Training_median_	0.40	0.98	−0.013	44	0.70	1.18	−0.161	28
Training_replicates_	0.34	1.03	0.339	40	0.64	1.27	−0.027	28

**Table 8:** Statistical measures of prediction performances obtained on SC-2 test sets with ANN using alternative descriptors for ALOGP.

100-Compound Test Set 1				32-Compound Test Set 2			
*R* ^2^	RMSE	Bias	% ± 0.5 Log	*R* ^2^	RMSE	Bias	% ± 0.5 Log
0.15	1.06	0.3	38	0.154	1.44	0.55	13

**Table 9:** Statistical measures of prediction performances obtained on SC-2 test sets with ANN based on 188 compounds of the training dataset.

Training Datasets	100-Compound Test Set 1				32-Compound Test Set 2			
	*R* ^2^	RMSE	Bias	% ± 0.5 Log	*R* ^2^	RMSE	Bias	% ± 0.5 Log
188-compound, without *T_mp_*	0.37	1.01	0.252	39	0.68	1.21	0.122	22
188-compound, with *T_mp_*	0.39	0.99	0.244	38	0.69	1.18	0.114	22

## Data Availability

The data presented in this study are available in the supplementary material of the paper.

## Supplementary Materials

The following materials are available online at https://www.mdpi.com/article/10.3390/pharmaceutics13071101/s1, Table S1: Training dataset, Table S2: Test set 1, Table S3: Test set 2.
